# Impact of Grape Seed Extract on Flavor and Functionality of Pea Protein Extrudates and Patties

**DOI:** 10.1111/1750-3841.71321

**Published:** 2026-07-28

**Authors:** Lily Lincoln, Wendy Lu, Audrey L. Girard

**Affiliations:** ^1^ Department of Food Science University of Wisconsin–Madison Madison Wisconsin USA

## Abstract

Consumer interest in plant‐based proteins is increasing, but undesirable off‐flavors from Maillard reaction and lipid oxidation processes hinder their broader acceptance. Our previous work showed that proanthocyanidin‐rich plant extracts like grape seed extract (GSE) could reduce the formation of these off‐flavors in aqueous plant protein solutions. This study investigated the effects of GSE addition pre‐ and post‐extrusion at two levels (0.02% and 1.0% w/w) on off‐flavor development in pea protein extrudates and patties. We hypothesized that GSE addition at different processing phases would impact its ability to alter Maillard reaction and lipid oxidation processes, thus affecting its ability to mitigate off‐flavor formation. Pre‐extrusion treatments reduced Maillard‐associated browning products by 40% more than post‐extrusion treatments, potentially due to greater protein–polyphenol interactions during extrusion‐induced protein denaturation and restructuring. Conversely, post‐extrusion GSE treatment decreased primary and secondary lipid oxidation products by approximately 53% and 44% in patties, respectively (vs. 15% and 31%, respectively, in pretreated samples), and enhanced antioxidant capacities across all samples. Because post‐extrusion GSE treatments exhibited fewer apparent protein–polyphenol interactions, a larger proportion of polyphenols may have remained chemically available for radical scavenging. Sensory evaluation suggests that GSE did not impart astringency, a common concern with polyphenol addition. These results indicate that polyphenols may be a promising strategy for improving flavor quality in plant‐based protein products.

**Practical Applications:**

Addition of grape seed extract can help reduce off‐flavors in legumes caused by major flavor degradation pathways. Adding polyphenols before processing can reduce Maillard‐derived off‐flavors, while polyphenols added after processing are more effective at reducing lipid oxidation‐derived off‐flavors. This approach offers a simple and sustainable way to enhance flavor quality and properties of plant‐based meat alternatives.

## 1 Introduction

Recently, consumer interest in plant‐based proteins has grown significantly, driven by their perceived sustainability, ethical considerations, and health benefits. In fact, 33% of US adults who eat meat report actively trying to reduce their meat and poultry consumption (Kirchner et al. 2021). As of 2025, the global plant‐based protein market is valued at approximately $24 billion USD and is projected to grow 7.9% between 2025 and 2030. Pea protein in particular is growing rapidly with a projected annual growth rate of 12.1% through 2030 (MarketsandMarkets 2025). However, broader consumer acceptance and continued growth are challenged by the presence of undesirable “beany” and “grassy” off‐flavors commonly associated with plant proteins (Kirchner et al. 2021). These off‐flavors are largely products of the Maillard reaction and lipid oxidation processes, both of which are promoted during cooking processes like extrusion.

Texturized proteins, such as pea protein extrudates, are a common ingredient in plant‐based meat alternatives because the extrusion process helps to develop a fibrous, chewy texture in plant proteins that is characteristic of meat. Extrusion achieves this by transforming the molecular structure of globular plant proteins through a combination of high heat, shear, and pressure. Throughout this process, proteins are exposed to conditions that accelerate the Maillard reaction and lipid oxidation (Plattner 2020).

Due to the susceptibility of plant proteins to flavor degradation during processes like extrusion that are necessary for textural development, interventions to slow or inhibit the Maillard reaction and lipid oxidation are needed. The Maillard reaction is a protein–carbohydrate conjugation reaction between the ε‐amino group of lysine in proteins and the terminal reducing carbonyl group of carbohydrates. While this process is important for the development of desirable flavors in traditional foods, it can also lead to the formation of off‐flavor compounds such as furans, pyrazines, pyridines, and aldehydes in plant protein products (El Hosry et al. 2025). Though most lipids are removed during the processing of protein isolates and concentrates, polar lipids such as phospholipids and free fatty acids remain. These lipids can oxidize during processing and storage, thus forming primary and secondary oxidation products such as peroxides, ketones, and saturated aldehydes, which contribute to grassy, bitter, and green flavors (Hadidi et al. 2022). Both reactions are intensified under the high temperature and shear conditions of extrusion.

Polyphenols, like those found in grape seeds, offer a potential solution to reduce off‐flavor formation (Lincoln and Girard 2025; Soendjaja and Girard 2024). Grape seeds, a byproduct of winemaking and juice production, are rich in polyphenols, including proanthocyanidins, flavan‐3‐ols, and phenolic acids, making them a promising value‐added ingredient for food applications. Proanthocyanidins are oligomeric and polymeric flavan‐3‐ols that are particularly potent antioxidants due to their multiple hydroxyl groups, which can scavenge free radicals and chelate metal ions (Rodríguez‐Pérez et al. 2019). The potent antioxidant effects and carbonyl‐trapping powers of polyphenol‐rich grape seed extract (GSE) have been shown to reduce undesirable lipid oxidation products and volatiles in aqueous plant protein systems (Soendjaja and Girard 2024). Furthermore, in precooked chicken breast, GSE inhibited the intensity of musty and rancid odors and flavors, supporting its general effectiveness as an antioxidant and off‐flavor mitigator (Brannan 2009). Additionally, GSE can bind to available amino groups on proteins in aqueous systems, limiting their interaction with reducing sugars and thereby slowing the Maillard reaction (Lincoln and Girard 2025). Collectively, these properties make GSE a promising functional ingredient for reducing off‐flavor formation in plant‐based protein products.

Building on these ideas, our study worked to close the gap between polyphenol interventions to reduce lipid oxidation and Maillard reaction processes in model aqueous systems and providing the same benefits in industry‐relevant extrudate production. We investigated the incorporation of GSE either before or after extrusion to determine its effectiveness in limiting off‐flavor development in extrudates and patties made from these extrudates. Extrudate samples were analyzed for key lipid oxidation and Maillard reaction products, then formed into patties to assess polyphenol functionality in a model food application. The most promising treatment was further evaluated through consumer sensory testing. This approach provides insight into how the timing of polyphenol addition influences their functionality in extrudates and their potential to improve flavor quality in plant‐based meat analogs.

## 2 Experimental Procedures

### 2.1 Materials

Pea protein isolate (PPI; 81.4% protein, db) and pea flour (PF; 55.9% protein, db) were donated by AGT (Regina, SK, Canada). Cornstarch was donated by Tate & Lyle (Rezista brand, London, UK). Wheat gluten from Bob's Red Mill (Milwaukie, OR), methylcellulose from Pure Original Ingredients (Solana Beach, CA), and pure refined coconut oil from Kroger (Cincinnati, OH) were purchased for patty formulation. GSE was donated by FutureCeuticals (Momence, IL). All other chemicals used in this study were of analytical grade. Deionized (DI) water was used to prepare all solutions.

### 2.2 Characterization of GSE

Hydrophilic interaction liquid chromatography (HILIC) was performed on GSE to quantify proanthocyanidins. Analysis was performed using a Dionex UltiMate 3000 high‐performance liquid chromatography (HPLC) system with an LPG‐3400 quaternary pump, a WPS‐300 analytical autosampler, a DAD‐3000 diode array detector, and an FLD‐3100 fluorescence detector (Thermo Fisher Scientific, Waltham, MA) with a Lichrosorb DIOL‐5 column, 5 µm, 250 mm × 4.0 mm (Supleco Analytical, Bellefonte, PA), based on the methods of Dorris et al. (2018). The samples were excited at 230 nm, and the emission at 321 nm was determined, with a sensitivity of 1. Two solvents were used to create a gradient: solvent A (98:2, v/v acetonitrile:acetic acid) and solvent B (95:3:2, v/v/v methanol:water:acetic acid). The gradient was set to 0–3 min, 0%–7% B; 3–60 min, 7%–37.6% B; 60–63 min, 37.6%–100% B; 63–70 min, 100% B; and 70–76 min, 100%–7% B; then equilibrated for 10 min at 7% B. The column oven was set to 35°C, the flow rate was 1 mL/min, and the injection volume was 20 µL.

### 2.3 Extrusion

**FIGURE 1:**
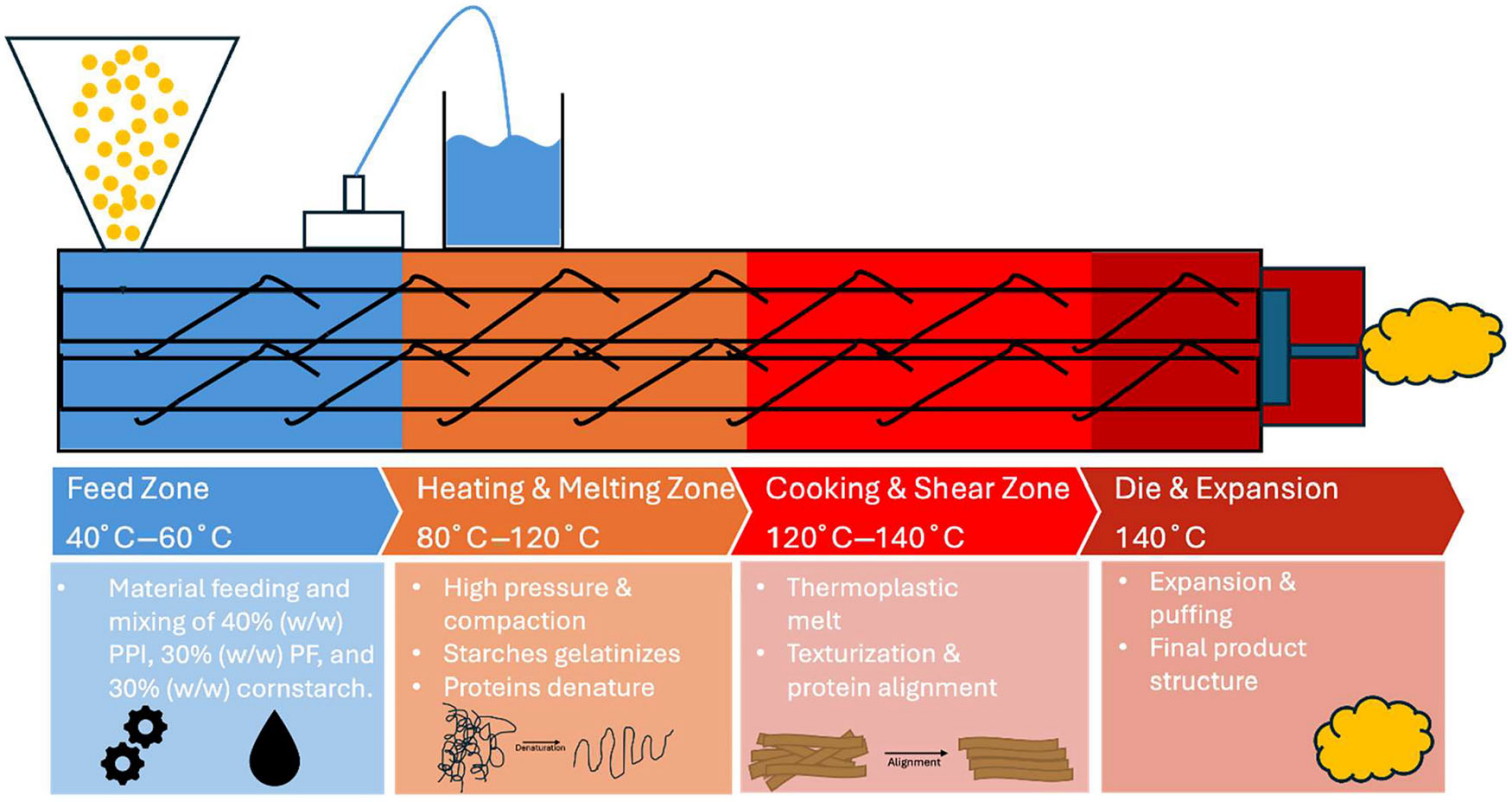
Schematic of the extrusion process used in this work. For the pre‐extrusion treatments, grape seed extract (GSE) was added via the water source. The dry ingredients were added at a feed rate of ∼500 g/h, and water was added at a feed rate of 190 mL/h.

A flow diagram of the extruder is shown in Figure 1. A ThermoFisher Scientific Process 11 Hygienic Parallel Twin‐screw Extruder was used to produce the extrudate for this study. The extruder was fixed with a standard intertwined screw design designed to create a low‐moisture texturized pea protein product. Eight temperature zones were set at 40°C, 60°C, 80°C, 100°C, 120°C, 140°C, 140°C, and 140°C, and the material was fed into a 6‐mm die, which was held at 140°C. The twin screw speed was kept constant at 450 rpm.

To evaluate polyphenols’ ability to mitigate off‐flavor formation, GSE was added pre‐ and post‐extrusion. GSE was chosen due to its high‐molecular‐weight polyphenols and their polymeric structure. GSE is a polyphenol extract derived from wine and juice‐making waste streams, making it lower in cost and more accessible and realistic for industry use as compared to isolated polyphenols, which are often expensive and difficult to source in large quantities (Franzen Ramos et al. 2023).

Two GSE levels (0.02% and 1.0% w gallic acid equivalent/w protein) were selected based on previous work and sensory considerations. The lower level was chosen based on previous work, which demonstrated GSE's ability to inhibit Maillard reaction and lipid oxidation pathways in aqueous protein systems (Lincoln and Girard 2025; Soendjaja and Girard 2024). The high level was selected based on similar extracts, such as green tea extract, becoming astringent and perceptible in extrudate‐based meat analogs when added above this threshold and evaluated by a trained sensory panel (Han et al. 2024).

The dry ingredients used for extrudate consisted of 40% (w/w) PPI, 30% (w/w) PF, and 30% (w/w) cornstarch. Pea protein was selected for its high market share, cost‐effectiveness, and desirability as an alternative to soy protein (MarketsandMarkets 2025). The dry ingredients were dispensed into the extruder using a gravimetric hopper at a dry feed rate of ∼500 g/h, and water was added at a feed rate of 190 mL/h. For samples with polyphenols added pre‐extrusion, 0.02% (pre‐lo) or 1.0% (pre‐hi) w/w of GSE was added into the extruder via the water source, as highlighted in Figure 1. The resulting extrudate product was dried in an oven at 40°C until the moisture content reached ∼10%. The extrudate was then ground using a Waring Commercial Heavy‐Duty Electric Spice Grinder (Stamford, CT) for 30 s. The material was stored at −20°C for further testing. Polyphenols added post‐extrusion were added to the ground extrudate at the same levels, 0.02% (post‐lo) or 1.0% (post‐hi), before storage.

### 2.4 Chemical and Physicochemical Characterization of Extrudate

#### 2.4.1 Protein–Polyphenol Interaction Analysis

A cold acetone precipitation was used to precipitate proteins and protein‐bound polyphenols, and then the free polyphenols in the supernatant were quantified. Ground extrudate samples were mixed with cold acetone (1:4 w/v). Samples were vortexed for 30 s and stored at −20°C for 1 h. Samples were centrifuged at 12,000 × *g* for 15 min to obtain the supernatant with free polyphenols. These free polyphenols were quantified using the Folin–Ciocalteu method (Singleton et al. 1999). Bound polyphenols were calculated by difference: amount of starting free polyphenols − amount of free polyphenol quantified after interacting.

Free lysine was determined as another indicator of protein–polyphenol interaction using a spectrophotometric, o‐phthalaldehyde (OPA) assay adapted from Tian et al. (2023). To make 100 mL of an OPA reagent, 80 mg OPA was dissolved in 2 mL ethanol, 1 mL sodium dodecyl sulfate, 200 µL 2‐mercaptoethanol, and 50 mL 0.1 M sodium borate buffer. DI water was then added to produce a total volume of 100 mL. Free amino acids were extracted by mixing 50 mg of ground extrudate with 3 mL of 10% trichloroacetic acid (TCA), reacting on ice for 30 min, and centrifuging at 10,000 × *g* for 10 min (Jin et al. 2022). A sample of 200 µL of the supernatant was mixed with 4 mL of OPA reagent, and the absorbance value was measured at 340 nm using a UV–vis spectrophotometer (Genesys 10S, Thermo Fisher Scientific). The standard curve was prepared using l‐lysine due to its reactivity and role as a key contributor to the Maillard reaction through protein glycation (Ashoor and Zent 1984).

#### 2.4.2 Determination of Antioxidant Capacity

To determine the antioxidant capacity of the extrudate, two methods were used: the Ferric Reducing Antioxidant Power (FRAP) assay and the 2,2′‐azino‐bis(3‐ethylbenzothiazoline‐6‐sulfonic acid) (ABTS) assay. FRAP measures the ability to reduce Fe^3+^ to Fe^2+^ (Apak et al. 2018), while ABTS measures the ability to quench radicals (Cano and Arnao 2018). Samples were extracted in a 1:40 (w/v) solution with 1% HCl in methanol for 2 h while shaking. The working reagents for FRAP and ABTS were added to 100 µL of the sample and incubated for 30 min at room temperature. The absorbance was measured at 593 nm for FRAP and 734 nm for ABTS. FRAP and ABTS values were quantified using ferrous sulfate and Trolox standards, respectively.

#### 2.4.3 Browning Index

At 420 nm, the browning index was used as an indirect measure of Maillard reaction‐associated browning products (Vélez et al. 2016). Briefly, 0.5 g of the extrudate was suspended in 10 mL of DI water. The solution was then homogenized (OMNI Mixer Homogenizer, OMNI International, Kennesaw). The homogenized samples were centrifuged at 12,000 × *g* for 10 min. The supernatant was filtered through Whatman Filter Paper #1, and the absorbance was read at 420 nm and standardized against GSE.

#### 2.4.4 Quantification of Lipid Hydroperoxide

The main product of lipid oxidation, lipid hydroperoxides (LOOH), was quantified using a modified version of the International Dairy Federation (Bligh and Dyer 1959; Shantha and Decker 1994). Lipids were extracted by adding 8 mL of chloroform:methanol (1:2 v/v) to a test tube containing 3 g of ground extrudate, followed by 10 s of vortexing. Then, 3 mL of chloroform and 2 mL of 0.5% KCl were added and vortexed for 10 s. The solutions were centrifuged at 12,000 × *g* for 10 min and filtered through Whatman Filter Paper #1. The lipids were then isolated into the chloroform (bottom) layer. A sample of 2 mL of this layer was mixed with 3 mL of a chloroform:methanol (7:3 v/v) solution, followed by the addition of 25 µL of iron (II) solution and 25 µL ammonium thiocyanate. Solutions were reacted for 5 min, after which absorbance was recorded at 500 nm. The peroxide value was calculated using a standard curve of iron (III) chloride solution.

#### 2.4.5 Quantification of Thiobarbituric Acid Reactive Substance (TBARS)

Secondary lipid oxidation products, including malondialdehyde (MDA) and other reactive substances, were quantified using the TBARS method (Vyncke 1975). MDA and other reactive substances were extracted by mixing 3.5 g of extrudate with 7.5 mL of 10% (v/v) TCA for 10 s, adding another 5 mL of TCA, and filtering the samples through Whatman Filter Paper #1. A sample of 2.5 mL of filtrate was mixed with thiobarbituric acid (46 mM in glacial acetic acid) and incubated in a 90°C water bath for 30 min. After cooling to room temperature, the absorbance was recorded at 532 nm.

#### 2.4.6 Water Holding Capacity (WHC)

To measure WHC, 0.5 g of ground extrudate was dispersed into 7.5 mL of water in a preweighed 15‐mL centrifuge tube. The mixture was then vortexed for 30 s, rested for 5 min, and centrifuged at 10,000 × *g* for 10 min. After centrifugation, the tube was inverted for 5 min and allowed to drain, and then the weight of the tube and remaining material was recorded (Ketnawa and Rawdkuen 2023). WHC was calculated as shown in Equation (1):

(1) $$\begin{array}{ccc} & & \mathrm{WHC}\;\left(\frac{g\;H_{2}O}{g\;\mathrm{sample}}\right)\\ & & \;=\frac{\mathrm{Weigh}t_{\mathrm{final}\;\mathrm{tube}}-\mathrm{Weigh}t_{\mathrm{empty}\;\mathrm{tube}}-\mathrm{Weigh}t_{\mathrm{ground}\;\mathrm{TVP}}}{\mathrm{Weigh}t_{\mathrm{ground}\;\mathrm{TVP}}}. \end{array}$$

#### 2.4.7 Expansion Ratio

The expansion ratio was calculated by dividing the average diameter of the extrudate (*D*_T_) by the die outlet diameter (*D*_D_), shown in Equation (2) (Maskus 2015). Three random measurements were taken of the diameter and length of the extrudate using a digital caliper (ThermoFisher Scientific).

(2) $$\mathrm{Expansion}\;\mathrm{ratio}=\frac{D_{T}}{D_{D}}$$

#### 2.4.8 Bulk Density

The bulk density (*p*, g/cm^3^) was calculated by dividing the mass of extrudate (*M*) by the volume it occupies (*V*), shown in Equation (3) (Lyu et al. 2022). The extrudate was weighed, and the length and diameter were measured using an electronic caliper. These values were used to calculate volume (*V*) using the equation below (Equation 4):

(3) $$p\left(\frac{g^{3}}{\mathrm{cm}}\right)=\frac{M}{V},$$

(4) $$V\;\left(cm^{3}\right)=\pi {\left(\frac{D}{2}\right)}^{2}\times L.$$

#### 2.4.9 Color Analysis

L*, a*, and b* values of extrudate were measured with a Hunter Lab colorimeter. Total color change (Δ*E*) values were calculated as follows (Lyu et al. 2022):

(5) $$\Delta E=\sqrt{\left[{\left(a^{*}-a_{0}^{*}\right)}^{2}+{\left(b^{*}-b_{0}^{*}\right)}^{2}+{\left(L^{*}-L_{0}^{*}\right)}^{2}\right]}.$$

### 2.5 Patty Formulation

Before the patties were formed, hydrated methylcellulose was prepared by blending 4 g of methylcellulose with 100 g of water in a Ninja Professional Plus Blender with Auto‐iQ (Needham, MA) for 1 min, followed by overnight hydration at 4°C. Marbling fat was prepared by blending 8 g of methylcellulose with 400 g of water, then slowly adding 1200 g of melted coconut oil until fully emulsified. This mixture was spread onto a sheet pan and frozen until solid. Extrudate was hand‐cut to a minimum size of 4 mm and a maximum size of 10 mm. The extrudate was rehydrated using boiling water (10:11 m/v), covered, and allowed to hydrate for 10 min.

To create the patties, rehydrated extrudate (63 g) was mixed with 6 g of wheat gluten, 9 g of PPI, 1.8 g of methylcellulose, and 0.5 g of salt. These ingredients were mixed with 9 g of a hydrated methylcellulose solution (dry methylcellulose and DI water [3:100 w/v], described above) and 10 g of shredded marbling fat (described above). The mixture was then allowed to rest for 20 min, after which the dough was formed into 30‐g patties measuring 0.5‐cm thick and 5 cm in diameter. The patties were baked at ∼191°C for 15 min, flipping the patties halfway through. The patties were completely cooled (∼1 h), then ground for 30 s using a Waring Commercial Heavy‐Duty Electric Spice Grinder (WSG30) for analysis.

### 2.6 Chemical and Physicochemical Analysis of Patty Product

#### 2.6.1 Protein–Polyphenol Interaction Analysis

The Folin–Ciocalteu method was used in accordance with the method described in Section 2.4.1. However, rather than acetone extraction, free polyphenols were extracted from the patty with 1% HCl in methanol for 2 h while shaking.

Free amino group content was quantified using the OPA method described in Section 2.4.1.

#### 2.6.2 Determination of Antioxidant Capacity

FRAP and ABTS assays were used as described in Section 2.4.2 to measure the reducing capacity of Fe^3+^ to Fe^2+^ and radical quenching ability, respectively.

#### 2.6.3 Browning Index

The browning index was used to measure the formation of the Maillard reaction indicators, as described in Section 2.4.3.

#### 2.6.4 Quantification of Lipid Hydroperoxide

LOOH was quantified using a modified version of the International Dairy Federation method as described in Section 2.4.4.

#### 2.6.5 Quantification of TBARS

To quantify secondary lipid oxidation products, the TBARS method was used as described in Section 2.4.5. MDA and other reactive substances were extracted by mixing with 3.5 g of patty sample and 15 mL of 10% TCA for 10 s, adding 10 mL of TCA, centrifuging at 12,000 × *g* for 10 min, and filtering the samples through Whatman Filter Paper #1.

#### 2.6.6 WHC

WHC was measured and calculated as described in Section 2.4.6.

#### 2.6.7 Cooking Yield

Cooking yield (%) was measured by weighing patties pre (Pr) and post (Po) baking. The % yield was calculated as follows:

(6) $$\mathrm{Cooking}\;\mathrm{yield}\;\left(\%\right)=\frac{\mathrm{Po}}{\mathrm{Pr}}\times 100.$$

#### 2.6.8 Color Analysis

L*, a*, and b* values of patties were measured and calculated in accordance with Section 2.4.9.

#### 2.6.9 Gas Chromatography (GC)

The volatile compounds from patty samples were analyzed using GC and mass spectrometry (MS). Compounds were extracted from a 1‐g ground patty sample using solid‐phase microextraction (SPME) on a 1‐cm, 24‐ga divinylbenzene/carboxen/polydimethylsiloxane (DVB/CAR/PDMS) fiber. The fiber was initially conditioned at 270°C for 30 min and then conditioned at 250°C for 10 min between uses. The sealed sample vial was held at 40°C for 20 min, and the volatiles were extracted on the fiber for 20 min. The fiber was injected into the GC port, and analysis was performed using an Agilent 6890N gas chromatograph (Agilent Technologies Inc., Palo Alto, CA) coupled to a mass‐selective detector (Agilent 5973 MS; Agilent Technologies Inc.). Analytes were desorbed from the SPME fiber for separation into a fused‐silica capillary column (RTx‐5MS, 30 m × 0.25 mm internal diameter, 0.5 µm film thickness; Restek Corp., Bellefonte, PA). Helium was used as the carrier gas at a 1.4 mL/min flow rate. The temperature began at 30°C for 5 min, then increased by 10°C/min until it reached 150°C. The mass spectrometer was operated at an ion source temperature of 230°C, an ionization voltage of 69.9 eV, and a mass scan range of *m*/*z* 33–250 at 6.17 scans/s. Recovered volatiles were identified by searching the National Institute of Standards and Technology (NIST) version 1.7 mass spectral database (Agilent Technologies Inc.).

### 2.7 Consumer Sensory Panel

The study was reviewed and approved by the University of Wisconsin–Madison Institutional Review Board (IRB) under approval #2021‐0865, and informed consent was obtained from each subject prior to their participation in the study. Consumers were recruited through email invitations and physical and online flyers from the Madison, WI, area (USA). Eligible participants for the study were at least 18 years old; had no allergy to legumes, wheat, or coconut; and predominantly consisted of students, faculty, and staff members of the UW‐Madison, as well as members of the public. Consumers were requested to complete a demographic screener prior to being served any samples, regarding age group, gender, and plant‐based meat analog consumption habits. Participants were compensated with assorted candy bars and bagged chips for completing the study, and each evaluation lasted approximately 15 min. Panelists were served three patties made with differing extrudates: commercial extrudate (Vestkorn Milling, Norway), lab control extrudate, and pre‐lo sample. A total of 75 participants completed the evaluation.

Panelists evaluated one sample at a time and were instructed to cleanse their palate with an unsalted cracker and a sip of water before evaluating each sample. Each consumer received three samples in a randomized and balanced presentation order; there were six possible orders presented (A–F). Patty samples were served warm (held in a warming cabinet at 60°C ± 4.0°C until served) within 15 min of cooking (Sogari et al. 2023). Patties were served on white paper plates labeled with a randomly assigned three‐digit code, along with a fork, an expectorant cup, crackers, and potable water on a plastic tray. Samples were assessed using a 9‐point hedonic scale for overall liking, flavor, and appearance (1 = *extremely dislike*, 9 = *extremely like*), in addition to 9‐point scales for the selected attributes of beaniness (1 = *not at all beany*, 9 = *extremely beany*), grassiness (1 = *not at all grassy*, 9 = *extremely grassy*), astringency (1 = *not at all astringent*, 9 = *extremely astringent*), and aftertaste (1 = *not like at all*, 9 = *like extremely*).

### 2.8 Statistical Analysis

Data analyses were performed using JMP Statistical Software (SAS Institute, Cary, NC). The treatment effects were determined by one‐way analysis of variance (ANOVA). Using Tukey's HSD, a 5% significance level was used to determine the effects between treatments. All treatments and chemical and physicochemical tests were run in triplicate. Sensory data were analyzed using a mixed‐model ANOVA including product treatment, serving order, and their interaction as fixed effects, with panelists included as a random effect. Tukey's HSD test was used for mean separation at a 5% significance level.

## 3 Results and Discussion

### 3.1 Polyphenol Composition of GSE

The GSE selected for this study had a high flavonoid content (127 mg/g powder), and its proanthocyanidins were 42.0% oligomeric and 19.3% polymeric (Table 1). Oligomeric and polymeric proanthocyanidins possess strong antioxidant activity and high affinity for protein binding due to their multiple available hydroxyl groups and relatively high molecular weight (Girard et al. 2018). This strong binding capacity enables interaction with free amino acids and reactive carbonyl species. Consequently, GSE has been shown to successfully inhibit flavor degradation in plant‐protein‐based aqueous systems (Lincoln and Girard 2025; Soendjaja and Girard 2024).

**TABLE 1:** Characterization of flavonoid content and degree of polymerization (DP) of grape seed extract used in this study.

	Grape seed extract
Flavonoid content (mg/g extract powder)	127 ± 0.2
DP 1–2 (%)	38.8 ± 2.4
DP 3–7 (%)	42.0 ± 6.9
DP 8+ (%)	19.3 ± 9.0

*Note*: Flavonoid content and DP measured using hydrophilic interaction liquid chromatography. Values are mean ± standard deviation.

### 3.2 Effects of Processing on Indicators of Pea Protein–Polyphenol Interactions

The extent of protein–polyphenol interactions in both extrudates and patties was evaluated using two complementary approaches: (1) quantification of free polyphenols following cold acetone precipitation using the Folin–Ciocalteu assay, with bound polyphenols calculated by difference, and (2) determination of free lysine content using the o‐phthaldialdehyde (OPA) method. Although the cold acetone precipitation is widely used to estimate protein‐associated phenolics, the Folin–Ciocalteu assay measures total reducing capacity rather than polyphenols specifically (Singleton et al. 1999). Thus, reducing compounds formed during cooking and extrusion, including Maillard reaction products, may contribute to the measured signals (Kitchen and Williamson 2024). Therefore, results should be interpreted as relative estimates of extractable versus protein‐associated reducing compounds rather than absolute measurements of polyphenol binding. To strengthen interpretation, Folin–Ciocalteu results were evaluated alongside OPA‐free lysine measurements, which independently supported increased protein–polyphenol interactions in pre‐extrusion treatments.

**FIGURE 2:**
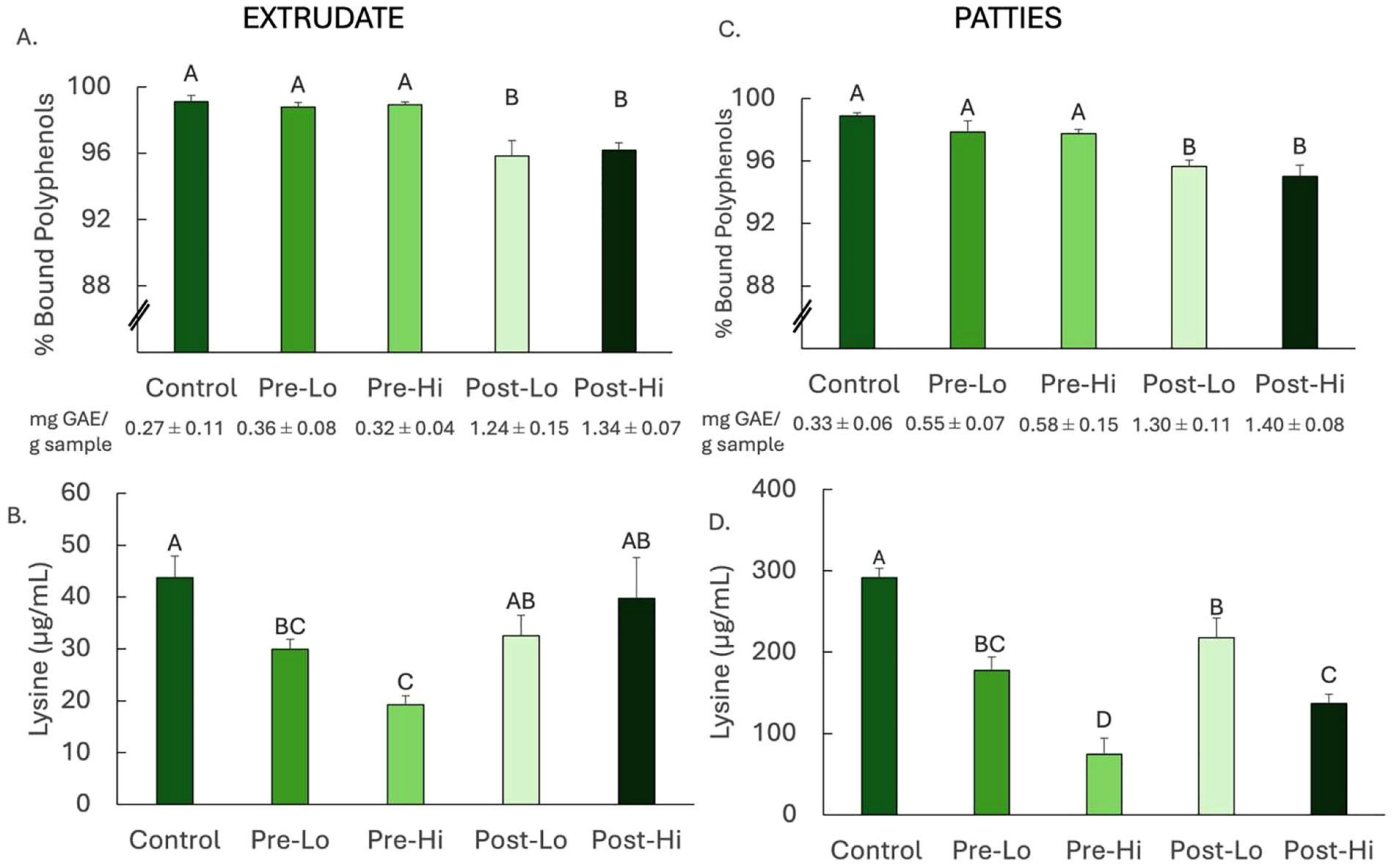
Effects of grape seed extract (GSE) treatments added pre‐ or post‐extrusion at 0.02% (‐lo) or 1.0% (‐hi) on indicators of protein–polyphenol binding. Percent of polyphenols bound to protein within extrudates (A) or patties (C). Free lysine content of extrudates (B) or patties (D). Different uppercase letters indicate significant differences (*p* < 0.05) between treatments.

In extrudates, the control treatment exhibited a Folin–Ciocalteu value of 0.27 ± 0.11 mg gallic acid equivalents (GAE)/g extrudate, indicating that the pea protein material had a low phenolic content and most of the present phenolic material was in a nonextractable form (Figure 2A). Pre‐lo and pre‐hi exhibited modestly higher values of 0.36 ± 0.08 and 0.32 ± 0.04 mg GAE/g extrudate, respectively, while post‐lo and post‐hi extrudates exhibited substantially higher extractable phenolic contents of 1.24 ± 0.15 and 1.34 ± 0.07 mg GAE/g extrudate, respectively.

Cooked patties formulated from the extrudates exhibited higher extractable phenolic contents than the extrudates themselves (Figure 2C). The control patty exhibited a Folin–Ciocalteu value of 0.95 ± 0.61 mg GAE/g patty, while pre‐lo and pre‐hi patties contained 0.55 ± 0.07 and 0.58 ± 0.15 mg GAE/g patty, respectively. Post‐lo and post‐hi exhibited the highest values, measuring 1.30 ± 0.11 and 1.40 ± 0.08 mg GAE/g patty, respectively. The Folin–Ciocalteu values observed in control samples likely reflect naturally occurring reducing compounds and endogenous phenolics inherent to pea protein, in line with other studies describing intrinsic phenolic and nonphenolic reductants in legume protein isolates (Faber et al. 2024). Further, the increase between extrudate and patties can be explained by the additional formation of Maillard reaction products and oxidized amino acids formed during cooking.

Pre‐extrusion treatments exhibited the greatest measured protein‐associated phenolic fractions, in both extrudate and patties (Figure 2A,C). Conversely, post‐extrusion exhibited significantly lower (*p* < 0.05) amounts of bound polyphenols. This observation aligns with established mechanisms of protein–polyphenol interactions, where polyphenols form both noncovalent (hydrogen bonding, hydrophobic, and electrostatic interactions) and covalent complexes with exposed amino acid side chains on proteins. Upon heating or mechanical disruption, tertiary structures are unfolded, exposing previously buried hydrophobic regions, in turn promoting protein–polyphenol interactions (Girard 2022; Hülsebusch et al. 2025). Because extrusion induces protein denaturation, aggregation, and formation of a thermoplastic melt, which can bury amino acid side chains, post‐extrusion GSE may have had limited opportunity for protein–polyphenol interactions (Hülsebusch et al. 2025). The reduction in bound polyphenols in patties compared to extrudates represents additional processing effects, polyphenol oxidation and polymerization, formation of insoluble complexes, and shifts in binding interactions (Song et al. 2023).

Free amino acid analysis revealed distinct trends in free lysine between extrudates and patties. In extrudates, pre‐extrusion treatments exhibited significantly lower (*p* < 0.05) free lysine concentrations compared to post‐extrusion treatments (Figure 2B), suggesting GSE may have reduced amino acid availability through protein–polyphenol interactions (Figure 2A). During extrusion, polyphenols can be oxidized, forming reactive quinones that readily react with nucleophiles, such as amino acids, including lysine, which contains a reactive epsilon‐amino group (─NH_2_) (Zhang et al. 2025). This epsilon group readily reacts with reducing sugars in the absence of polyphenols, further promoting the Maillard reaction. However, with the presence of pre‐extrusion added polyphenols, these quinones may have interacted with the reactive lysine groups, which is consistent with the significant decrease (*p* < 0.05) in free lysine (Figure 2B,D).

In cooked patties, all treatments showed significantly lower (*p* < 0.05) free lysine levels relative to the control (Figure 2D), with no significant differences observed among the low‐level treatments. Although patties were formulated from the extrudates, free lysine concentrations were higher in patties than in the corresponding extrudates. This increase is attributable to the incorporation of untreated PPI and wheat gluten during patty formulation, which contributed additional lysine to the system. Overall, pre‐extrusion treatments exhibited the greatest protein‐associated phenolic fractions and the largest reductions in free lysine throughout extrusion and cooking, indicating pre‐extrusion incorporation may be more effective at promoting protein–polyphenol binding.

### 3.3 Effects of GSE on the Antioxidant Capacity of Extrudates and Patties

**FIGURE 3:**
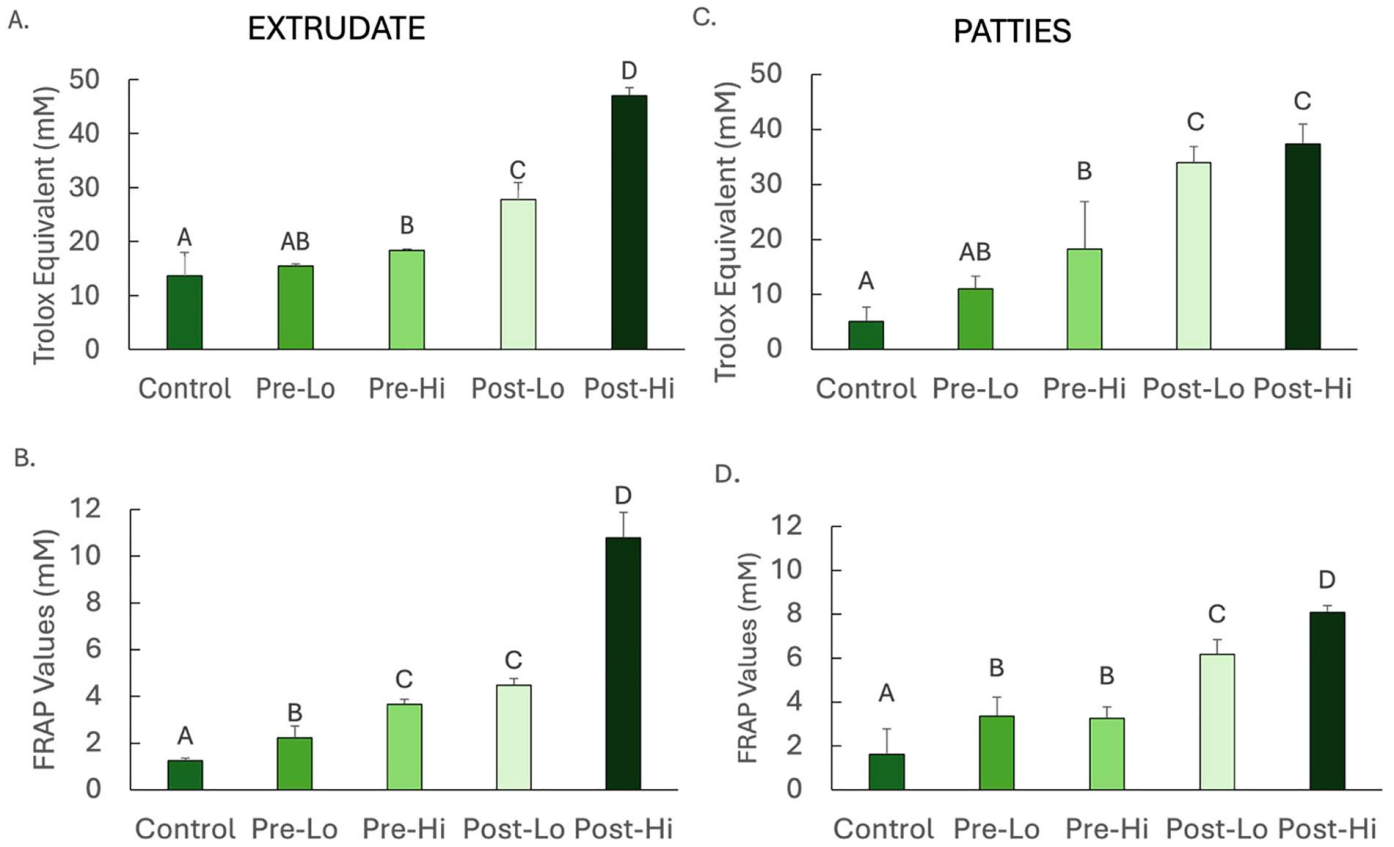
Effects of grape seed extract (GSE) treatments added pre‐ or post‐extrusion at 0.02% (‐lo) or 1.0% (‐hi) on antioxidant capacities. The radical scavenging abilities of extrudates (A) and patties (C) were measured using ABTS. The ferric reducing power of extrudates (B) and patties (D) was measured using the FRAP assay. Different uppercase letters indicate significant differences (*p* < 0.05) between treatments.

Antioxidant capacity of extrudate and cooked patty samples was assessed using FRAP and ABTS assays. In extrudates, post‐hi treatment exhibited the greatest reducing capacity and the highest quenching power (Figure 3A,B). This was likely because polyphenols added post‐extrusion remained more extractable and chemically available for electron or hydrogen donation to neutralize free radicals (Wu et al. 2024; Zhangli et al. 2025). In contrast, pre‐lo and pre‐hi exhibited significantly lower (*p* < 0.05) ABTS and FRAP values, consistent with lower concentrations of free polyphenols (Figure 2A). During extrusion, protein denaturation and restructuring may have promoted protein–polyphenol interactions, reducing the accessibility of polyphenols for radical scavenging despite their retention within the system (Y. Feng et al. 2023; Wei et al. 2025).

In cooked patties, all treatments except pre‐lo showed a significant increase (*p* < 0.05) in ABTS values compared to the control (Figure 3C). Similarly, FRAP values significantly increased (*p* < 0.05) for all treatments (Figure 3D). Interestingly, patty antioxidant activity measured by FRAP and ABTS decreased by approximately 30% and 23%, respectively, in the post‐hi treatment compared to the corresponding extrudate values. Despite this decrease, post‐lo and post‐hi maintained the greatest antioxidant capacities in patties, indicating that post‐extrusion addition preserved a greater amount of extractable and functionally active polyphenols. In contrast, the lower antioxidant capacities observed in pre‐treated samples are consistent with reduced polyphenol accessibility following protein–polyphenol interactions during extrusion. Similar decreases in polyphenol extractability during thermal processing have been reported in sorghum bran tortillas throughout mixing, baking, and storage (Dunn et al., 2015).

### 3.4 Effects of GSE on Maillard Browning of Extrudates and Patties

**FIGURE 4:**
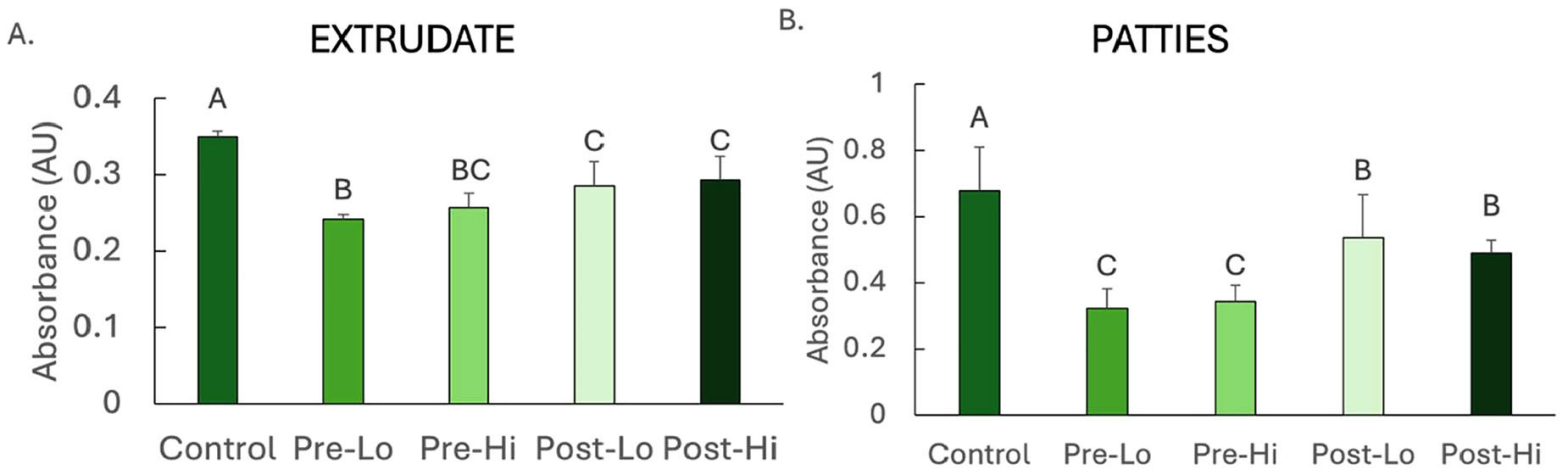
Effects of grape seed extract (GSE) treatments added pre‐ or post‐extrusion at 0.02% (‐lo) or 1.0% (‐hi) on brown pigment in extrudates (A) and patties (B). Measured by absorbance at 420 nm. Different uppercase letters indicate significant differences (*p* < 0.05) between treatments.

The browning index test was used to provide an indirect measurement of thermal browning associated with the Maillard reaction (Figure 4). Pre‐hi extrudate was expected to show the largest reduction in browning products due to a higher polyphenol content and increased protein–polyphenol interactions (Figure 2A,B). Surprisingly, pre‐lo exhibited the greatest reduction in browning, at 31% compared to 27% in the pre‐extrusion high level (Figure 4A). The observed increase in absorbance in the pre‐hi sample may be explained by the thermal reactivity of GSE, which can undergo oxidation and polymerization upon heating, forming brown quinone pigments that absorb around 420 nm (Nieto et al. 2024). Thus, formation of GSE‐degradation pigments may have contributed to the higher‐than‐expected browning index observed in the pre‐hi and potentially masked reductions in Maillard‐derived browning. Post‐extrusion addition also resulted in a significant reduction (*p* < 0.05) in browning products, likely due to the increased antioxidant capacity of the polyphenols (Figure 3A,B), which can scavenge residual carbonyls responsible for further browning (Peng et al. 2010).

The cooked patty control samples showed a 39% increase in browning index compared to the extrudate control (Figure 4B), as expected due to further processing and heating. Among the cooked patty treatments, pre‐lo and pre‐hi exhibited the greatest suppression of browning end products. Pre‐lo resulted in browning index values approximately 40% lower than post‐lo, while pre‐hi GSE showed a ∼30% reduction relative to the corresponding post‐hi (Figure 4B).

Overall, pre‐treated samples exhibited a greater reduction in browning index than post (Figure 4). This trend may be attributed to greater opportunities for protein–polyphenol interactions during extrusion, when protein denaturation and restructuring expose reactive amino residues, potentially reducing the availability of key Maillard reaction sites through binding interactions (Figure 2B,D) (Yin et al. 2014). Conversely, polyphenols in the post‐treated samples were likely limited to interactions with surface‐accessible regions, explaining the smaller reduction in browning index.

### 3.5 Effects of GSE on the Lipid Oxidation Products of Extrudates and Patties

**FIGURE 5:**
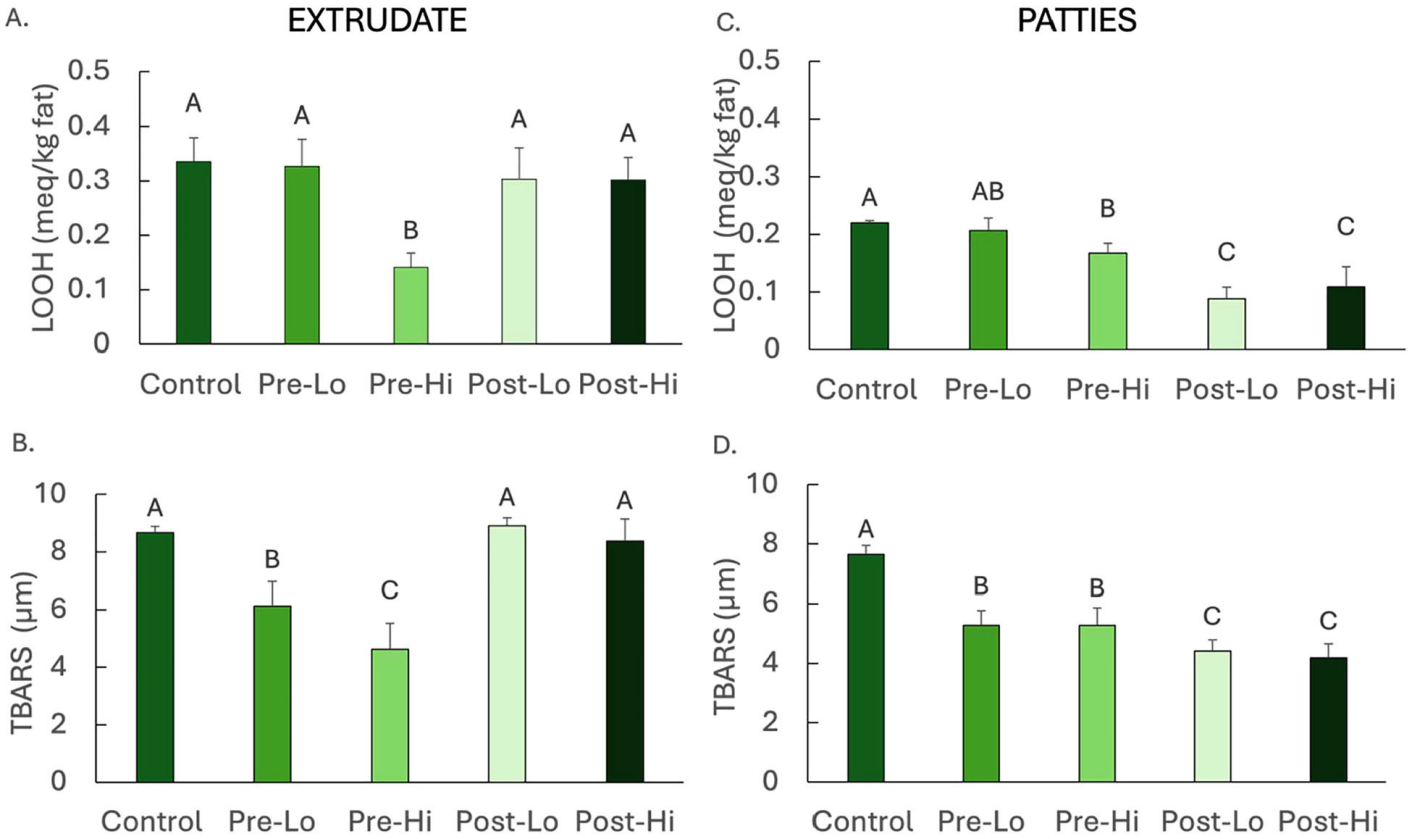
Effects of grape seed extract (GSE) treatments added pre‐ or post‐extrusion at 0.02% (‐lo) or 1.0% (‐hi) on lipid oxidation products. Hydroperoxides (LOOH) were quantified as primary lipid oxidation products of extrudates (A) and patties (C). Thiobarbituric acid reactive substances (TBARS) were quantified as secondary oxidation products of extrudates (B) and patties (D). Different uppercase letters indicate significant differences (*p* < 0.05) between treatments.

Lipid oxidation was evaluated by measuring primary and secondary oxidation products (LOOH and TBARS, respectively). LOOH levels within the controls decreased by approximately 65% from extrudate to cooked patties (Figure 5A,C), reflecting the intrinsic instability of hydroperoxides. The weak O─O bond in LOOH promotes rapid thermal decomposition during cooking, shifting oxidation toward secondary products. At advanced oxidation stages, hydroperoxide decomposition exceeds formation, resulting in lower detectable LOOH (Nollet and Toldrá 2009).

In the extrudates, only the pre‐hi treatment significantly reduced (*p* < 0.05) LOOH formation (Figure 5A), indicating that suppression of primary oxidation was dependent on both timing and concentration. In patties, the pre‐lo treatment again showed no significant reduction (*p* > 0.05), whereas pre‐hi and both post‐extrusion treatments significantly reduced peroxide values (Figure 5C). LOOH formation occurs rapidly during the initiation and propagation phase of oxidation when lipid radicals react with oxygen to form lipid peroxyl radicals that generate LOOH. Effective inhibition of LOOH requires antioxidants to kinetically outcompete propagation and be present at sufficiently high concentration in the lipid phase (Valgimigli 2023). Therefore, treatments that maintained higher concentrations of active phenolics during processing would be expected to more effectively suppress primary oxidation products such as LOOH.

With respect to secondary lipid oxidation products in the extrudates, both pre‐lo and pre‐hi significantly reduced (*p* < 0.05) TBARS relative to the control, whereas post‐extrusion treatments had no significant effect (Figure 5B). In cooked patties, all treated groups exhibited significantly lower (*p* < 0.05) TBARS compared to the control, with post‐lo and post‐hi producing the greatest reductions (60% and 50.6%, respectively; Figure 5D). The results are consistent with the hypothesis that post‐extrusion addition more effectively preserves antioxidant activity. Because GSE added post‐extrusion was not exposed to the high heat and shear conditions of extrusion, flavan‐3‐ols and procyanidins may have retained greater antioxidant activity due to reduced thermal degradation (Figure 3A,B). Moreover, phenolic binding data (Figure 2) suggest that pre‐extrusion incorporation promoted greater protein–polyphenol interactions, which may have reduced polyphenol accessibility and antioxidant activity. In contrast, post‐lo and post‐hi retained higher levels of free polyphenols, enhancing their capacity to donate a hydrogen atom and chelate transition metal ions, two primary mechanisms by which antioxidants terminate lipid oxidation (Figure 3C,D) (Wu et al. 2024). Interestingly, the pre‐lo GSE treatment did not significantly reduce LOOH formation (Figure 5A) but reduced secondary oxidation products (Figure 5B). Secondary oxidation products form later through the decomposition of LOOH into secondary carbonyl compounds. Secondary product formation requires a longer timescale, giving low‐concentration antioxidants more opportunity to suppress formation through radical scavenging and carbonyl trapping (Abeyrathne et al. 2021; Valgimigli 2023).

### 3.6 Effects of GSE on the Physical Properties of Extrudates and Patties

#### 3.6.1 Color Analysis

Color measurements indicated significant effects of GSE addition on extrudates and cooked patties (Tables 2 and 3; Figure S1). In extrudates, posttreatments were significantly lighter (*p* < 0.05) than the pretreatments (Table 2), likely due to polyphenol oxidation and polymerization during extrusion in the pretreated samples (Murata 2022). Pre‐hi exhibited more red hues (higher a* value) than pre‐lo, reflecting early Maillard reaction products and thermally degraded polyphenols (Murata 2022). Posttreated samples had higher yellow pigmentation by comparison because their polyphenols remained intact and retained a higher antioxidant capacity (Figure 3A,B), which suppressed oxidative and browning interactions on the surface of the extrudate (Figure 4). Although significant differences (*p* < 0.05) were observed in the individual L*, a*, and b* values, reflecting chemical changes within the extrudates, no significant differences were detected in Δ*E* between samples, indicating that no single treatment caused a markedly larger overall color change. Nevertheless, all samples exhibited Δ*E* values greater than 3, suggesting that the color differences would still be perceptible (Pathare et al. 2013).

**TABLE 2:** Effects of grape seed extract (GSE) treatments on color parameters (L*, a*, b*, Δ*E*), expansion ratio, and bulk density of extrudates.

Treatment	L*	a*	b*	Δ*E*	Expansion ratio	WHC (g H_2_O/g)	Bulk density (g/L)
Control	30.4 ± 6.1^A^	4.94 ± 1.5^A^	16.5 ± 1.9^AB^	NA	1.22 ± 0.2^A^	1.68 ± 0.1^A^	1.14 ± 0.2^A^
Pre‐lo	27.4 ± 4.9^A^	4.15 ± 0.2^B^	14.2 ± 0.6^BC^	4.25 ± 2.0^A^	1.16 ± 0.9^A^	1.78 ± 0.0^A^	0.76 ± 0.1^B^
Pre‐hi	29.1 ± 4.1^A^	4.87 ± 0.4^A^	13.0 ± 1.3^C^	4.13 ± 3.2^A^	1.22 ± 0.2^A^	1.78 ± 0.1^A^	0.62 ± 0.1^B^
Post‐lo	34.5 ± 1.9^B^	3.44 ± 0.4^B^	16.5 ± 0.8^AB^	5.06 ± 0.8^A^	1.19 ± 0.1^A^	2.45 ± 0.1^B^	1.23 ± 0.5^A^
Post‐hi	34.6 ± 3.6^B^	4.65 ± 0.7^AB^	16.7 ± 1.4^A^	4.65 ± 3.5^A^	1.14 ± 0.1^A^	2.76 ± 0.1^C^	1.25 ± 0.2^A^

*Note*: Values are mean ± standard deviation. Different superscript letters within each column indicate significant differences among treatments (*p* < 0.05). Pre‐ versus post‐ indicates addition of GSE before or after extrusion, while ‐lo versus ‐hi indicates 0.02% or 0.1% w GSE/w protein, respectively.

**TABLE 3:** Effects of grape seed extract (GSE) treatments on color parameters (L*, a*, b*, Δ*E*), water holding capacity (WHC), and cooking yield of extrudate‐based patties.

Treatment	L*	a*	b*	Δ*E*	WHC (g H_2_O/g)	Cooking yield (%)
Control	52.2 ± 2.9^A^	15.7 ± 3.2^A^	38.0 ± 1.6^AB^	NA	0.61 ± 0.1^A^	76.5 ± 2.5^A^
Pre‐lo	54.2 ± 2.2^A^	14.9 ± 2.2^A^	39.5 ± 2.5^A^	6.24 ± 0.6^A^	0.88 ± 0.04^AB^	81.7 ± 2.5^B^
Pre‐hi	46.9 ± 1.0^B^	13.2 ± 2.7^A^	29.6 ± 4.3^B^	15.7 ± 3.8^B^	0.86 ± 0.1^AB^	83.3 ± 1.0^B^
Post‐lo	52.0 ± 1.4^AB^	15.6 ± 2.7^A^	39.9 ± 2.3^A^	7.03 ± 0.1^A^	0.99 ± 0.2^B^	81.0 ± 0.5^B^
Post‐hi	49.2 ± 0.3^AB^	11.3 ± 2.3^A^	30.6 ± 1.4^B^	13.8 ± 0.5^B^	0.99 ± 0.2^B^	82.0 ± 0.4^B^

*Note*: Values are mean ± standard deviation. Different superscript letters within each column indicate significant differences among treatments (*p* < 0.05). Pre‐ versus post‐ indicates addition of GSE before or after extrusion, while ‐lo versus ‐hi indicates 0.02% or 0.1% w GSE/w protein, respectively.

In cooked patties, pre‐hi was the only treatment significantly darker (*p* < 0.05) than the control (Table 3), consistent with polyphenol oxidation and protein–polyphenol complex formation (Figure 2) (Murata 2022). Both pre‐ and post‐hi treatments exhibited significantly lower b* values (*p* < 0.05), reflecting either pigment formation or polyphenol‐mediated suppression of carbonyl formation and nonenzymatic browning (Tongnuanchan et al. 2011). All samples exhibited significant Δ*E* values (Δ*E* > 3), indicating noticeable color changes (Pathare et al. 2013), with the pre‐hi and post‐hi treatments showing the largest overall color differences. This significant change is not inherently negative, as the presence of darker brown pigments can contribute to desirable pigmentation that mimics meat in plant products (Ryu et al. 2023). Therefore, the color changes observed may offer functional advantages, in addition to reducing undesirable degradation products (Figures 4 and 5), by enhancing the visual appeal of plant‐based meat mimetics.

#### 3.6.2 Bulk Density of Extrudates

Extrudate pretreated samples had significantly lower (*p* < 0.05) bulk densities than the control and post‐extrusion samples (Table 2), indicating that pretreated extrusion samples may have higher porosity despite no significant differences in expansion ratio. Similar relations have been reported in milk proteins extruded with fruit pomace powders, where internal porosity and density varied independently of expansion (Iqbal and Rizvi 2023).

#### 3.6.3 WHC of Extrudates, and WHC and Cooking Yield of Patties

WHC was significantly higher (*p* < 0.05) in post‐extrusion samples compared to control and pre‐extrusion treatments in both extrudates and patties (Tables 2 and 3), likely because more free hydrophilic polyphenols were available to interact with and bind water. Cooking yield increased (*p* < 0.05) by ∼7% across all polyphenol treatments compared to the control (Table 3). This suggests that pre‐extrusion polyphenols can still improve yield, likely through matrix stabilization, even though they do not significantly enhance WHC. Post‐extrusion polyphenols, on the other hand, provide ∼6% more free phenolics (Figure 2) that can interact with water, contributing to increased WHC and cooking yield. WHC and cooking yield are both key determinants of eating quality in patties because they influence moisture retention, juiciness, and texture during consumption. WHC reflects the ability of the protein matrix to retain water during cooking and eating. Higher WHC has been directly associated with improved juiciness and lower cooking losses, both of which enhance sensory appeal in meat and meat analog products (Warner 2023).

### 3.7 Effects of GSE on Off‐Flavor Volatile Compounds in Patties

**FIGURE 6:**
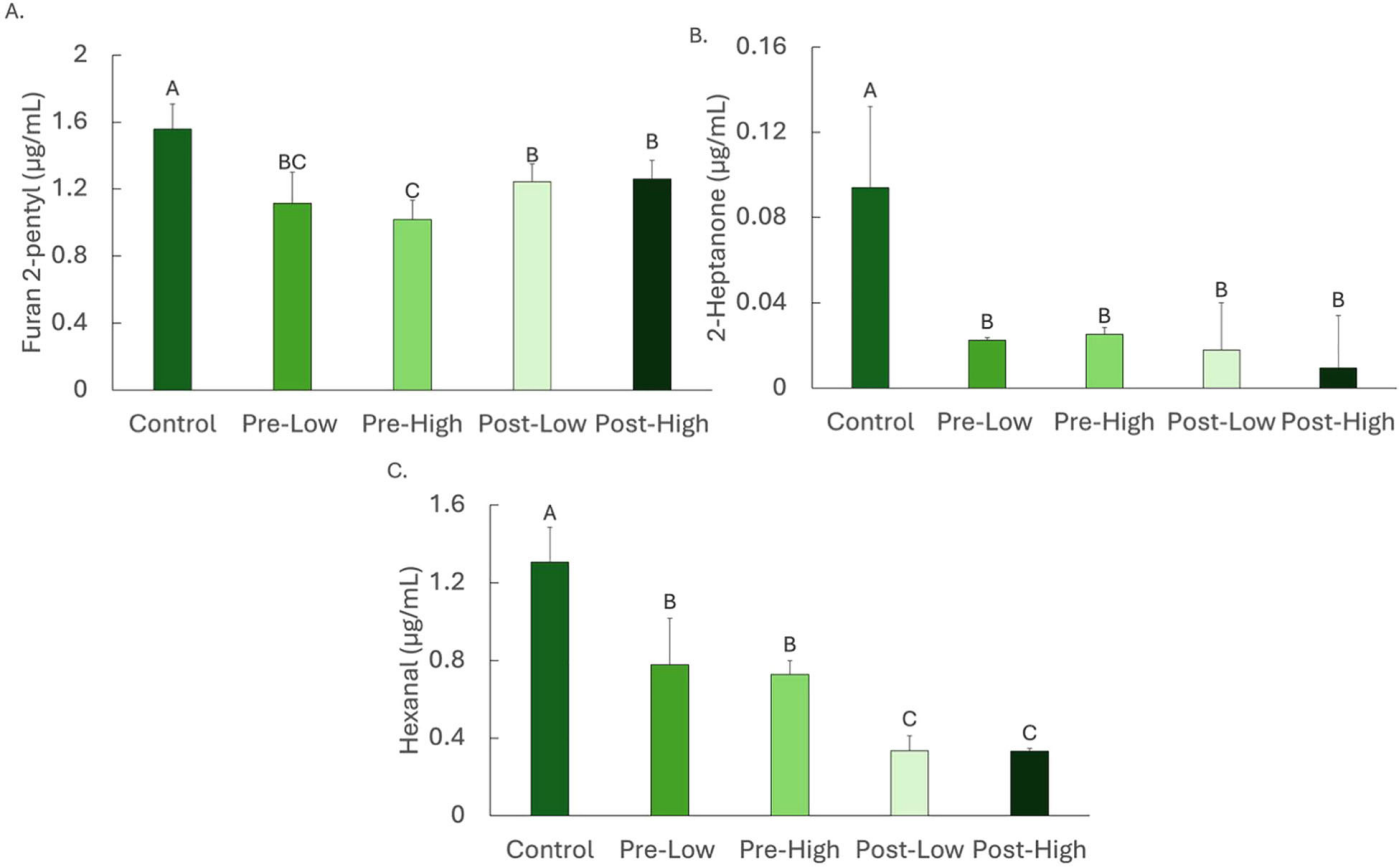
Effects of grape seed extract (GSE) treatments added pre‐ or post‐extrusion at 0.02% (‐lo) or 1.0% (‐hi) on the formation of volatiles furan 2‐pentyl (A), 2‐heptanone (B), and hexanal (C) within patties. Different uppercase letters indicate significant differences (*p* < 0.05) between treatments.

GC was used to quantify impactful flavor volatiles associated with lipid oxidation and Maillard reaction pathways in cooked patties. Through untargeted MS analysis, the major off‐flavor volatiles identified included furan 2‐pentyl, hexanal, and heptanone (Figure 6). Both pre‐ and post‐additions significantly reduced furan 2‐pentyl concentrations (*p* < 0.05; Figure 6A). Pre‐lo and pre‐hi were particularly effective, lowering furan 2‐pentyl by an average of 11.9% more than post‐extrusion addition. This brought levels close to the flavor threshold of 1 µg/mL (Krishnamurthy et al. 1967); pre‐low was 1.1 ± 0.2 µg/mL, and pre‐high was 1.01 ± 0.1 µg/mL versus the control at 1.56 ± 0.15 µg/mL. Furan 2‐pentyl imparts beany and grassy flavors and is formed via oxidation of linoleic acid and through the Maillard reaction by degradation of Strecker aldehydes, which are generated by the reaction of α‐dicarbonyls and amino acids (Li et al. 2019; Zheng et al. 2015). Potential polyphenol binding to reactive lysine residues may limit proteins’ ability to interact with reducing sugars, thus slowing the rate of the Maillard reaction and reducing the formation of volatile compounds such as furan 2‐pentyl. The enhanced lysine binding (Figure 2B,D) and greater reduction of browning index values (Figure 4) in pretreatments support this mechanism.

2‐Heptanone, a secondary lipid oxidation product formed from the decomposition of hydroperoxides and subsequent rearrangement into methyl ketones, was significantly reduced (*p* < 0.05) by all treatments (Figure 6B) (Grebenteuch et al. 2021). Similar rates of suppression among samples were unexpected, as 2‐heptanone is formed via oxidation, whose rate of reduction is heavily reliant on antioxidant capacity (Grebenteuch et al. 2021). Pre‐lo treatment significantly suppressed (*p* < 0.05) 2‐heptanone despite its inability to reduce LOOH (Figure 5C) and limited quenching power (Figure 3A). Protein‐bound polyphenols may have intercepted radicals and stabilized intermediates prior to degradation. Pre‐lo samples exhibited moderate FRAP (Figure 3C), indicating a reducing power sufficient to quench lipid intermediates and reduce 2‐heptanone formation (Y. Feng et al. 2023). In contrast, posttreated samples, characterized by higher levels of free polyphenols (Figure 2C) and enhanced antioxidant capacities (Figure 3C,D), effectively limited 2‐heptanone formation, likely through radical scavenging and metal chelating activity.

Hexanal, a highly reactive primary aldehyde formed by lipid oxidation via hydrogen abstraction and β‐scission reactions, was significantly reduced (*p* < 0.05) by all polyphenol treatments (Figure 6C) (Feng et al. 2022). While the reduction in 2‐heptanone was uniform across treatments (Figure 6B), post‐extrusion treatments were more effective at suppressing hexanal than pre‐extrusion treatments (Figure 6C). This difference can be explained by the intrinsic chemical stability of the two volatiles. 2‐Heptanone is a secondary ketone, and its carbonyl group is stabilized by two electron‐donating groups, making it less reactive and less prone to degradation. Aldehydes, like hexanal, possess a more electrophilic carbonyl carbon and lack steric stabilization, making them more susceptible to oxidation (McClements and Decker 2017). This increased reactivity makes hexanal particularly sensitive to antioxidant protection. Consequently, post‐extrusion treatments, with higher antioxidant capacity (Figure 3) and greater levels of free phenolics (Figure 2), were the most effective at suppressing hexanal formation. Hexanal is associated with grassy, green, and cardboard‐like off‐flavors in foods. In this study, measured hexanal concentrations were 1.31 ± 0.18 µg/mL in the control, 0.78 ± 0.24 µg/mL for pre‐lo, 0.73 ± 0.07 µg/mL for pre‐hi, 0.33 ± 0.08 µg/mL for post‐lo, and 0.33 ± 0.01 µg/mL for post‐hi, which clearly shows the significant reduction achieved by post‐lo and post‐hi treatments.

### 3.8 Consumer Sensory Evaluation of GSE‐Treated Patties

Sensory evaluation was conducted to determine the impact of pre‐lo GSE treatment on the flavor, texture, and overall acceptability of extrudate‐based patties. Pre‐lo was selected because it successfully reduced volatile formation, inhibited secondary lipid oxidation products, and reduced browning associated with the Maillard reaction (Figures 6, 5, and 4, respectively). Additionally, pre‐lo treatment was less likely to contribute to astringent off‐flavors common with GSE (Habschied et al. 2021). No significant differences (*p* > 0.05) were observed among samples for astringency or aftertaste (Table 4), indicating that the incorporation of GSE did not introduce any exogenous off‐flavors relative to the control (Sabra et al. 2021).

**TABLE 4:** Sensory evaluation of plant‐based patties with pre‐extrusion low (pre‐lo) grape seed extract (GSE) treatment compared to experimental and commercial controls.

Patty treatment	Beaniness	Astringency	Aftertaste	Liking
Commercial control	4.17 ± 1.9^B^	2.65 ± 1.8^A^	3.85 ± 2.0^A^	4.36 ± 0.2^B^
Experimental control	4.64 ± 2.1^AB^	2.69. ± 1.8^A^	4.27 ± 2.1^A^	4.99 ± 0.2^A^
Pre‐Lo	5.12 ± 1.9^A^	2.69 ± 1.9^A^	3.81 ± 1.7^A^	4.86 ± 0.2^A^

*Note*: Attributes assessed include beaniness, astringency, aftertaste, and overall liking, scored on a standardized scale (1 = *no X*, 9 = *extreme X*). Values are mean ± standard deviation (*n* = 75). Different superscript letters within each column indicate significant differences among treatments (*p* < 0.05).

Interestingly, beaniness was significantly higher (*p* < 0.05) in the pre‐lo patty compared to the commercial control, while no significant difference (*p* > 0.05) was observed between commercial and experimental controls. This was unexpected because the pre‐lo treatment had been shown to significantly reduce (*p* < 0.05) volatiles responsible for beany characteristics in GC analysis (Figure 6). Further analysis of the sensory data indicated that a significant sequence effect (*p* < 0.05) was present, suggesting a first‐sample bias where the first‐tasted sample tended to receive higher beaniness ratings (Figure S2). Adjusted means showed that pre‐lo was perceived as most beany when served first (5.49 ± 0.338) and slightly lower when following experimental or commercial controls (5.05 ± 0.37 and 4.814 ± 0.39, respectively), demonstrating a trend consistent with contrast effects where the perceived intensity of a sample decreases when it is tasted after another sample with similar or higher flavor intensity (Mazur et al. 2018). Similar order‐dependent trends were observed for the experimental and commercial controls, with beaniness ratings varying according to tasting position. Although the only statistically significant (*p* < 0.05) pairwise positional difference was the decrease in experimental control from first to third order, the significant overall sequence effect indicates that tasting order consistently influenced perception across products, and the lack of statistically significant interaction (*p* > 0.05) between product and serving order indicates that all samples were affected equally. Moreover, panelist differences accounted for approximately 16% of the total variability, demonstrating that individual perception also contributed meaningfully to beaniness ratings.

Additional sensory bias may have arisen from distraction effects as the commercial control was noticeably different in appearance and texture, with consumers noting it was more “granular,” “lumpy,” and “spongy” (Figure S3). Visual and textural differences are known to influence flavor perception (Zampini and Spence 2012) and may have reduced detectable differences between experimental control and pre‐lo samples. Overall liking showed no significant difference (*p* > 0.05) between the experimental control and pre‐lo, indicating no change in palatability induced by GSE. Despite having a significantly lower perceived beaniness flavor from the pre‐lo sample, the commercial control received a significantly lower (*p* < 0.05) overall liking score. This further supports the influence texture and appearance had on the flavor perception of the samples.

Overall, sensory evaluation suggested that GSE incorporation did not reduce overall acceptability, while data discussed previously showed GSE effectively reduced chemical products associated with oxidation. Further sensory evaluation of extrudates containing GSE added both pre‐ and post‐extrusion is warranted to better determine whether the treatments consistently mitigate off‐flavors or whether contextual effects influenced perceptible differences. Future evaluations could limit distraction error by more closely standardizing sample appearance, texture, and size, while utilizing a trained sensory panel may also provide more precise evaluation of specific flavor attributes by reducing panelist variability (Ares and Varela 2017).

## 4 Conclusions

GSE added pre‐ and post‐extrusion at generally low levels (<1%) reduced indicators associated with lipid oxidation and Maillard reaction product formation in plant‐based extrudates and patties. Pre‐extrusion treatments exhibited an affinity for apparent protein–polyphenol binding, resulting in a more prominent reduction of browning products associated with the Maillard reaction. Post‐extrusion samples retained higher levels of free polyphenols, and exhibited enhanced antioxidant capacity along with greater reductions in the primary and secondary products of lipid oxidation and their volatiles. Sensory evaluation suggests that low levels of GSE can be added during extrusion and impart no further off‐flavors or aftertaste. These results confirm that GSE may be a viable option for mitigating undesirable off‐flavors common in plant‐based products and improving consumer acceptability. Future work should focus on obtaining direct mechanistic evidence of the proposed flavor degradation inhibitory effects of GSE added pre‐ and post‐extrusion. Additionally, future work is warranted to evaluate GSE functionality across broader formulations and processing conditions that mimic industrial products. Lastly, future studies should employ a more extensive sensory study that limits distraction errors, while further investigating the influence of polyphenol types, levels, and processing conditions on product quality and consumer perception.

## Author Contributions

**Lily Lincoln**: data curation, formal analysis, investigation, methodology, writing – original draft, conceptualization. **Wendy Lu**: conceptualization, writing – original draft, formal analysis. **Audrey L. Girard**: supervision, conceptualization, funding acquisition, project administration, resources, methodology, writing – review and editing.

## Conflicts of Interest

The authors declare no conflicts of interest.

## Supporting information




**Supplementary Figures**: jfds71321‐sup‐0001‐Figures.docx
