# Supplementary material for: Impact of Grape Seed Extract on Flavor and Functionality of Pea Protein Extrudates and Patties
Source: J Food Sci. 2026 Jul 28;91(7):e71321. doi: 10.1111/1750-3841.71321 (PMC13413622; doi:10.1111/1750-3841.71321)
Supplement: Supplementary file 1 — Supplementary Figures: jfds71321‐sup‐0001‐Figures.docx [file JFDS-91-0-s001.docx]

**
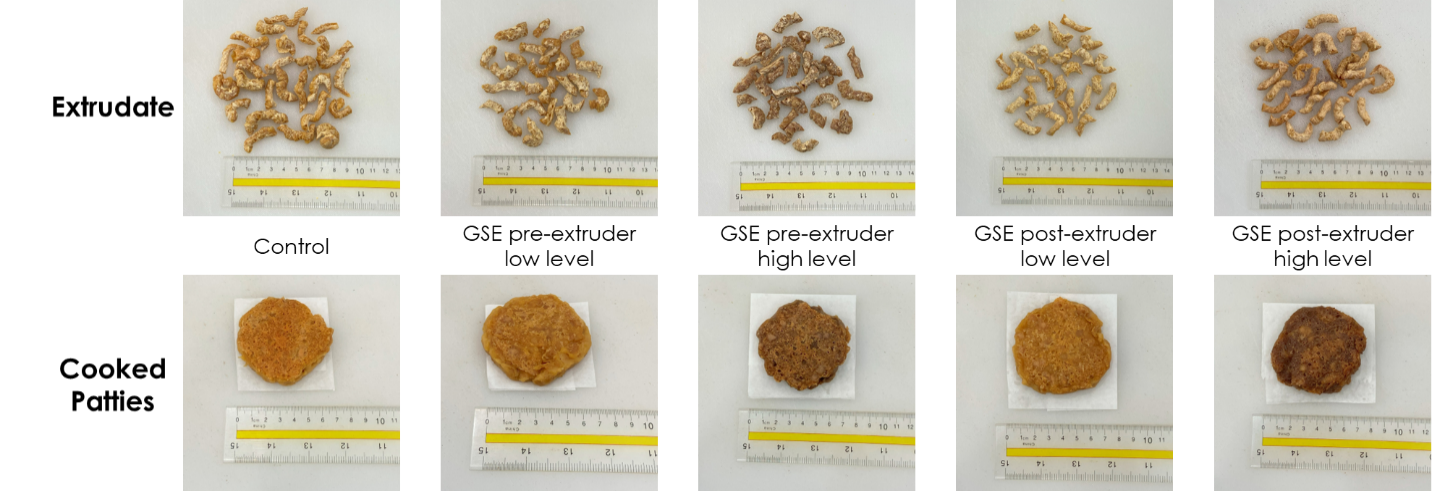
**

**Fig. S1** Images of extrudates. The control and post-extruder treated extrudates do not have added GSE, thus show the natural variation within pea protein extrudates.

**Fig. S2** Beaniness ratings of extrudate-based patties (124 = pre-lo GSE, 216 = commercial control, 857 = experimental control) across tasting positions (1 = first, 2 = second, 3 = third). Adjusted least-square means ± standard error is shown. Serving order had a significant overall effect (p < 0.05). A significant contrast effect (* = p < 0.05) was observed for 857 between the first and third positions. No other product × sequence contrasts were statistically significant (p > 0.05)

**Fig. S3.** Photographs of extrudate-based patties used in the sensory evaluation: 124 = pre-lo GSE treatment, 857 = experimental control, 216 = commercial control. Visual differences in color, texture, and surface appearance are evident, with sample 216 appearing more granular and “lumpy” compared to 124 and 857. These visual and textural differences may have influenced flavor perception, contributing to a distraction effect in sensory ratings.
